# Pedagogic prestidigitation: using magic tricks to enhance educational videos

**DOI:** 10.7717/peerj.9610

**Published:** 2020-07-21

**Authors:** Richard Wiseman, William Houstoun, Caroline Watt

**Affiliations:** 1 School of Psychology and Sport Science, University of Hertfordshire, Hatfield, Hertfordshire, UK; 2 Centre for Performance Science, Royal College of Music, University of London, London, UK; 3 School of Philosophy, Psychology and Language Sciences, University of Edinburgh, Edinburgh, UK

**Keywords:** Magic, Conjuring, Education, Online, Psychology

## Abstract

Previous research suggests that magic tricks can be employed within an educational context to enhance attention, engagement, critical thinking and recall. This study builds on this work by examining the impact of incorporating magic tricks into an online educational video. Adult participants (*N* = 198) completed a need for cognition scale and then watched a video containing either several bespoke card tricks that had been specially devised to help tell the story of the Apollo Moon landings (Magic Video), or an almost identical video that did not contain any magic tricks (Control Video). All participants rated their levels of engagement, absorption and recall. Compared to the Control Video, the Magic Video was rated as significantly more interesting, informative and absorbing. There was no difference between the groups for recall. There was a positive correlation between participants’ need for cognition scores, and the degree to which they found the Magic Video interesting, and were willing to share it with others. The theoretical, methodological and practical implications of these results are discussed, along with recommendations for future work.

## Introduction

Many educational practitioners have described using magic tricks to help promote attention, engagement, curiosity and critical thinking (Vidler & Levine, 1981; Frith & Walker, 1983; McCormack, 1985, 1990; Broome, 1995). Wiseman & Watt (2020) recently reviewed studies that have assessed the educational potential of magic tricks and concluded that much of the work yielded positive effects. However, their review also noted that it was problematic to draw strong conclusions from the literature because many of the studies suffered from various methodological issues, including a lack of control conditions, small sample sizes and poorly designed interventions. Wiseman and Watt concluded by making recommendations for future work in this area, including urging researchers to incorporate control groups, and to develop and employ bespoke magic-based interventions that are designed in collaboration with experienced magicians.

As well as having practical implications, the work may help to inform two quite different theoretical perspectives.

First, a large amount of work into key epistemic emotions, such as surprise and curiosity, has consistently shown that such emotions are positively associated with increased levels of knowledge engagement in both adults (Vogl et al., 2019a, 2019b; Pekrun, 2019) and infants (Spelke, 1988, 1991; Baillargeon, 1994). Magic tricks have been shown to be a highly effective way of generating both curiosity and surprise (e.g., Parris et al., 2009; Danek et al., 2015; Ozono et al., 2020) and so are likely to be especially effective at promoting knowledge engagement. This notion is supported by a study reported by Subbotsky (2010), in which participants were shown a seemingly impossible event being presented as either an example of magic or a new form of technology. Both children and adults exhibited more exploratory behavior and curiosity in the ‘magic’ condition.

Second, other research has examined the effect of ‘seductive details’ (i.e., material that is, interesting but irrelevant to instructional material) on learning (for reviews, see Rey, 2012; Sundararajan & Adesope, 2020). This work suggests that such details can often act as a distraction and have a detrimental effect on subsequent recall. Seen from this perspective, the inclusion of magic tricks in an educational video could be viewed as a seductive detail that would negatively impact on knowledge retention. However, other research suggests that this effect may not hold for material presented in a magical context. Subbotsky & Matthews (2011) asked both adolescents and adults to watch advertisements that either did, or did not, contain seemingly magical effects (i.e., talking animals or objects suddenly appearing). No differences emerged on an immediate free-recall test, but in a recognition test adolescents identified significantly more of the ‘magical’ advertisements. A total of 2 weeks later, adults, rather than adolescents, showed increased recognition of the “magical” films.

The vast majority of the studies reviewed by Wiseman and Watt involved magic tricks being presented in ‘live’ settings, such as talks, lectures, performances and demonstrations. Although these types of face-to-face interactions are likely to remain important in the future, the increased accessibility and scalability of web-based content has resulted in an increasing amount of educational material being offered online. Given this trend, it’s perhaps surprising that only one study has explored the impact of using magic tricks to enhance online education.

Moss, Irons & Boland (2017) carried out an online study involving just over 200 adult participants. One group of participants watched a video of a magic trick, a second group watched a video of a circus act, and a third group didn’t watch either video. Half of the participants who had watched the magic trick were told the secret of the illusion whilst the other half were not. All of the participants then completed a need for cognition scale (reflecting the extent to which they enjoy thinking and problem solving; Petty et al., 2008) and watched a video tutorial about neuroscience. In the final part of the study, participants completed a questionnaire designed to measure how absorbed they were in the tutorial and their retention of key information from the tutorial. The results suggested that: (i) watching the magic trick, but not finding out the solution to the illusion, lowered participants’ need for cognition scores; (ii) watching either the magic trick or the circus act lowered participants’ level of absorption in the tutorial; and (iii) participants’ memory for the tutorial did not differ across the groups. Moss, Irons & Boland (2017) argued that not knowing the secret to the magic trick may have distracted participants and interfered with their subsequent levels of absorption. They also acknowledged that the results may have been influenced by the nature of the Magic Video used in the study (a gory version of the classic sawing in half illusion), because the footage was both shocking and unrelated to the content of the tutorial.

The current study further examined whether the inclusion of magic tricks can help to boost viewers’ engagement, absorption and recall. In doing so, it aimed to both help educational practitioners produce better online materials, and to contribute to theoretical discussions about the impact of ‘seductive details’ and epistemic emotions on learning. The study built on the Moss, Irons & Boland (2017) study in two ways. First, rather than using a magic trick that was unrelated to the educational material, the study employed a series of bespoke tricks that were specifically designed to highlight the educational messaging. Second, the study explored how participants’ need for cognition was related to the perception and recall of the magic-based intervention. This variable was chosen because a large body of education-based research suggests that it correlates with engagement, learning and knowledge retention (Evans, Kirby & Fabrigar, 2003; Cazan & Indreica, 2014). In addition, need for cognition has been shown to be affected by watching magic tricks (Moss, Irons & Boland, 2017), and strongly relates to the cognitive elements that underpin many magic tricks (Rensink & Kuhn, 2015).

Prior to the study, we created two videos about the fiftieth anniversary of the Apollo Moon landings. Both videos had exactly the same educational narrative and were based around a specially created deck of playing cards that featured images of astronauts, space rockets, etc. In one video, several magic tricks were employed to create surprising and baffling moments during the narrative (Magic Video), whilst in the other the cards were simply dealt from the deck (Control Video). Following the methodological guidelines recommended by Wiseman & Watt (2020), these bespoke videos were carefully matched and created in collaboration with an experienced magician. During the study, adult participants first completed a need for cognition scale and watched either the Magic Video or the Control Video. Participants then rated their level of engagement and absorption, and attempted to recall key facts presented in the video.

It was predicted that participants would find the Magic Video more engaging, absorbing and memorable than the Control Video. In addition, it was predicted that participants’ need for cognition scores would positively correlate with engagement, absorption and recall in both conditions, and that these correlations would be significantly higher among those who had watched the Magic Video compared to those who had watched the Control Video.

## Materials and Methods

### Participants

Participants (*N* = 202, mean age = 30.45, range 18–65 years) were recruited from the crowdsourcing platform Prolific Academic. The use of these types of platforms for psychological research has been validated in several studies (Crump, McDonnell & Gureckis, 2013; Enochson & Culbertson, 2015). It wasn’t possible to estimate an expected effect size in advance of the study due to the lack of previous research in the area. However the predetermined sample size had a high chance of detecting a medium effect (*d* = 0.5, *p* < 0.05, 2-tailed, power = 0.9).

### Stimulus videos

Magicians have long used decks of playing cards to tell stories (for an historical overview of this approach see Behr, 2020), and we adopted this approach to create two stimulus videos about the Apollo Moon landings.

The first and second authors both have a background in magic, and the second author has won various accolades for sleight of hand performances. The two of them worked together to produce a video (Magic Video) that consisted of a soundtrack containing key facts about the Moon landings and several magic tricks performed with a playing cards containing relevant images. For instance, at the start of the video the narrator explained that the race to the Moon began in 1957, when the Soviets launched the world’s first satellite, Sputnik. At this point, the date “1957” suddenly appeared on a previously blank playing card, and moments later another card with an image of the Sputnik satellite magically emerged from the middle of the deck. The video lasted 1 min 50 s, and involved a static shot of a performer’s hands manipulating the cards above a black tablecloth. A version of the video was posted online prior to the experiment as part of the Moon landings 50th anniversary celebrations.

The magic tricks used to illustrate the story were chosen to use easily recognisable objects (playing cards and a coin) and to feature a series of fourteen impossible moments over the course of the video. The illusions were chosen to feature a variety of effects (including productions, transformations, animations and transpositions) that were developed by combining and adapting existing magic techniques to suit the required moments in the narrative (Neve, 1716; Downs, 1909; Pierce, 1909; Scott, 1909; Stanyon, 1912; Rosencrance, 1924; Elliot, 1953).

A second video (Control Video) contained the same audio narration as the Magic Video but did not contain any magic tricks. Instead, the cards were simply dealt from the pack and placed onto the table. The video was closely matched to the Magic Video, in that it contained the same playing cards, displayed each for the same amount of time, had the same duration, and also involved a static shot of playing cards being manipulated on a black tablecloth. The videos are available in Supplemental Material.

### Need for cognition scale

The NCS-6 is a reliable and valid short measure of need for cognition (De Holanda Coelho, Hanel & Wolf, 2018). It involves six statements (e.g., “I would prefer complex to simple problems”) with participants responding to each statement on a scale between “1” (Very uncharacteristic of me) and “5” (Very characteristic of me). Participants’ scores were averaged across the six items.

### Engagement items

Participants were asked to rate on a 5-point Likert scale (i) how interesting they found the video (1: not very interesting, 5: very Interesting), (ii) the degree to which the video had made them interested in the Apollo Moon landings (1: not very interested, 5: very Interested), (iii) how entertaining they found the video (1: not very entertaining, 5: very entertaining), (iv) how informative they found the video (1: not very informative, 5: very informative), (v) how likely they were be to share the video with others (1: very unlikely to share, 5: very likely to share), and (vi) the degree to which the video made them feel as if anything were possible (1: definitely disagree, 5: definitely agree). Participants’ scores on each item were treated as separate variables.

### Absorption questionnaire

This measure was devised and used by Moss, Irons & Boland (2017), and based on the absorption subscale of the Schoolwork Engagement Inventory (Salmela-Aro & Upadaya, 2016). It consisted of three statements (e.g., “While watching the video, time seemed to fly”), with participants rating their level of agreement to each item on a scale between “1” (Definitely Disagree) to “5” (Definitely Agree). Participants’ scores were averaged across the three items.

### Recall questionnaire

Participants were presented with six questions about key factual information presented in the videos (e.g., “According to the video, when was the Sputnik satellite launched?”) along with five possible responses for each question (e.g., “1955”, “1956”, “1957”, “1958”, “Cannot remember”). The correct answer was assigned 1 point, and participants’ scores were averaged across the six items.

No other measures were administered or data collected.

### Procedure

The study received ethics approval (number 10-1920/1) from the University of Edinburgh PPLS Research Ethics Committee. Participants were recruited on the Prolific Academic crowdsourcing platform, and the study was presented via Qualtrics. After giving written informed consent, participants were asked to enter their age and complete the NCS-6. They were then randomly assigned to watch either the Magic Video or the Control Video. After watching the appropriate video, participants were asked to indicate whether they had seen the video before (“Yes”, “No”, “Maybe”). Participants then completed the Engagement Items, the Absorption Questionnaire and the Recall Questionnaire. The time taken for each participant to complete the survey was recorded (in seconds), and they received $2.00 for taking part.

## Results

Four participants indicated that they may have seen the Magic Video before, and so were excluded from the analyses (final cohort: *N* = 198, mean age = 30.60, range 18–65 years). There were 99 participants in the Magic Video condition, and 98 in the Control Video condition. The two groups did not differ in mean age (Magic Video age = 30.79 years, SD = 11.61; Control Video age = 30.40, SD = 8.87; *t*_195_ = 0.26, *p* = 0.80), mean NCS-6 scores (Magic Video NCS-6 = 19.93, SD = 4.54; Control Video NCS-6 = 20.83, SD = 4.23; *t*_195_ = −1.43, *p* = 0.15), or mean time taken to complete the study (Magic Video time = 428.18 s, SD = 257.47; Control Video = 424.29 s, SD = 229.97; *t*_195_ = 0.11, *p* = 0.91).

Unpaired *t*-tests were used to compare the scores obtained from the two groups on the Engagement Items, the Absorption Questionnaire and the Recall Questionnaire. Compared to the Control Video, the Magic Video was rated as significantly more interesting, entertaining and informative. In addition, the Magic Video obtained significantly higher scores on the Absorption Questionnaire. There was no difference between the two groups on the Recall Questionnaire (see Table 1).

**Table 1:** Summary data for the Magic Video compared to the Control Video.

	Magic Video mean (SD)	Control Video mean (SD)	*t*	*p*	*d* (95% CI)
How interesting?	3.96 (0.99)	3.45 (1.08)	3.45	**0.0007**	0.49 [0.21–0.77]
How interested in Moon landings?	3.45 (0.87)	3.25 (1.05)	1.40	0.15	0.20 [− 0.07 to 0.49]
How entertaining?	4.03 (0.97)	3.5 (1.03)	3.72	**0.0003**	0.53 [0.48–1.06]
How informative?	3.86 (0.93)	3.55 (0.94)	2.31	**0.02**	0.33 [0.19–0.75]
How likely to share?	2.78 (0.92)	2.71 (0.93)	0.48	0.63	0.07 [−0.2 to 0.35]
Feel anything were possible?	3.31 (1.04)	3.05 (1.12)	1.70	0.09	0.24 [−0.04 to 0.52]
Absorption Questionnaire	3.43 (0.86)	3.08 (0.84)	2.93	**0.004**	0.42 [0.13–0.69]
Recall Questionnaire	4.28 (1.34)	4.52 (1.07)	−1.43	0.15	−0.20 [−0.48 to 0.08]

**Note:**

Means, SDs (in parentheses), unpaired *t*-values (df = 195), *p*-values (2-t; significant *p*-values in bold), and effect sizes (Cohen’s *d*; 95% Confidence Intervals in parentheses) for participants watching the Magic Video (*N* = 99) compared to the Control Video (*N* = 98).

To assess the relationship between the participants’ need for cognition and engagement, absorption and recall, Pearson correlations were calculated between NCS-6 scores and each of these variables in both conditions (see Table 2). For the Magic Video, need for cognition was positively related to how interesting participants found the video and the likelihood of them sharing it with others. For the Control Video, need for cognition was positively related to recall.

**Table 2:** Correlations between participants’ NCS-6 scores and each of the variables for the Magic Video and the Control Video.

	Magic Video	Control Video
How interesting?	0.21 [0.01–0.39] **(0.04)**	0.04 [−0.16 to 0.24] (0.66)
How interested in Moon landings?	0.10 [−0.10 to 0.29] (0.34)	0.006 [−0.19 to 0.20] (0.95)
How entertaining?	0.14 [−0.06 to 0.33] (0.17)	0.06 [−0.14 to 0.26] (0.55)
How informative?	0.13 [−0.07 to 0.32] (0.19)	0.03 [−0.17 to 0.23] (0.73)
How likely to share?	0.22 [0.02–0.40] **(0.03)**	0.01 [−0.19 to 0.21] (0.91)
Feel anything were possible?	0.13 [−0.07 to 0.32] (0.19)	0.002 [−0.20 to 0.20] (0.98)
Absorption questionnaire	0.13 [−0.07 to 0.32] (0.19)	0.06 [−0.14 to 0.26] (0.54)
Recall questionnaire	0.12 [−0.08 to 0.31] (0.25)	0.22 [0.02–0.40] **(0.03)**

**Note:**

Pearson correlations between participants’ NCS-6 scores (95% Confidence Intervals and 2-t *p*-values in parentheses; significant *p*-values in bold) and each of the variables among those watching the Magic Video (*N* = 99) and the Control Video (*N* = 98).

## Discussion

This study examined the impact of incorporating magic tricks into an educational video. Participants either watched a video in which magic tricks were used to highlight key facts about the Apollo Moon landings (Magic Video), or a very similar video that did not contain any tricks (Control Video). Participants then completed measures of engagement, absorption and recall. Compared to the Control Video, the Magic Video was rated as significantly more entertaining, informative and interesting. The Magic Video was associated with significantly higher levels of absorption, and there was no difference between the two videos for recall. There was a positive correlation between participants’ need for cognition and the degree to which they found the Magic Video interesting and were willing to share it with others. Finally, there was a positive correlation between participants’ need for cognition and recall for the Control Video. These findings will be discussed in turn.

First, the finding that the Magic Video was rated as more entertaining, informative, interesting and absorbing, supports previous work suggesting that magic tricks can play a positive and practical role in education (for a review, see Wiseman & Watt, 2020). From a more theoretical perspective, this finding further supports the notion that epistemic emotions, such as curiosity and surprise, have a significant and positive impact on knowledge engagement.

The high absorption ratings for the Magic Video runs contrary to the results obtained by Moss, Irons & Boland (2017), who reported that watching a magic trick lowered absorption levels. However, whilst their study employed a trick that was both relatively shocking and unrelated to a subsequent educational tutorial, our tricks were carefully designed to highlight educational messaging. As such, the findings highlight the importance of practitioners and researchers working with experienced magicians to create magic-based interventions that are carefully crafted to emphasise educational messaging.

Future work on this topic could explore the variables that may play a key role in the creation of effective magic-based interventions, including the nature of the magic tricks, the way that they relate to educational messaging, and the impact of performing style. Additional work could also examine how this research could help to inform theoretical debates surrounding the impact of important epistemic emotions, such as curiosity and surprise. As noted above, the ability to design interventions that will help operationalize these variables will benefit from working with an experienced and skilled magician. Additional work could also explore how the efficacy of such magic-based interventions compares to other techniques commonly used within education, including whiteboard animations, Prezi, Powerpoint and oral presentations (Turkay, 2016; Moulton, Turkay & Kosslyn, 2017). Finally, future work could also examine whether this use of magic tricks causes viewers to associate the topic of the video with greater positive affect, and whether they are motivated to engage in additional knowledge exploration outside of viewing the video.

Second, participants watching the Magic Video and Control Video did not differ in their ability to recall key facts from the videos. This finding is in line with similar results reported by Moss, Irons & Boland (2017) and supports previous work showing that the inclusion of “seductive details” doesn’t enhance the subsequent recall of key material (Rey, 2012; Sundararajan & Adesope, 2020). Future work could examine why the magic tricks failed to have the predicted impact on recall and attempt to design more effective magic-based videos. This work could, for instance, involve using eye-tracking studies and self-report methodologies to explore how this use of magic tricks impacts on viewers’ ongoing attention and thinking. Additional work could explore the effect of delivering factual information during a moment of magic (e.g., a key date suddenly appearing on a blank playing card) versus presenting the information outside of such moments. Finally, both this study, and Moss, Irons & Boland (2017), tested recall for relatively straightforward factual material almost immediately after participants had watched the video. Future work could examine the impact that magic tricks might have on the recall of more subtle information, and over a longer time period.

Finally, for the Magic Video participants’ need for cognition was positively correlated with their interest in the video and willingness to share it with others. This is in line with previous work showing that need for cognition is associated with greater engagement with educational material (Evans, Kirby & Fabrigar, 2003; Cazan & Indreica, 2014), and suggests that magic-based videos may be especially attractive for those who enjoy thinking and problem solving. Interestingly, participants’ need for cognition was positively correlated with recall for the Control Video but not for the Magic Video. In reality, this apparent difference may be illusory as an analysis, albeit exploratory and relatively low powered, revealed that the two correlations were not significantly different from one another (*z* = −0.71, *p* = 0.24). However, if the effect is genuine, the lack of a significant correlation for the Magic Video may be due to a form of “attention residue” (Leroy, 2009), wherein an uncompleted task (in this case, not being able to solve a magic trick) becomes a distraction and impairs attention and recall for subsequent activities. In the context of magic and education, the “attention residue” hypothesis was originally proposed by Moss, Irons & Boland (2017) and this topic could also be explored in future research. This work could, for instance, examine the impact of variables that are likely to be associated with need for cognition, such as the ease with which the magic tricks can be solved.

On a methodological level, the study was designed to adhere to many of the recommendations made by Wiseman & Watt (2020) in their review of magic tricks within a pedagogic setting. Wiseman and Watt urged researchers to employ control conditions and to work with magicians to create bespoke magic-based interventions. In the current study, the Magic Video and Control Video were very closely matched, and it is hoped that this approach could provide a template for future work in the area. In addition, the magic-based intervention was created in collaboration with an experienced, widely read and skilled magician, and carefully crafted to highlight key educational messages. This work illustrates the benefits of such partnerships, and it is hoped that other research in the area will adopt a similar approach.

## Conclusions

Overall, this work extends the findings from other studies examining the positive impact of magic tricks (Wiseman & Watt, 2018; Bagienski & Kuhn, 2019). Within an educational setting, the current study shows that such tricks have the potential to enhance online educational videos by boosting engagement, interest and absorption. In addition, the findings showed that the inclusion of magic tricks did not cause any loss of recall, and the videos appeared to be especially attractive to individuals with a higher need for cognition. Unlike much of the previous work in the area, the current study employed a tightly matched control condition and involved collaborating with an experienced magician to create a bespoke magic-based intervention. It is hoped that future work will build on these findings and continue to explore the expansive and educational potential of magic.

## Supplemental Information

10.7717/peerj.9610/supp-1 Supplemental Information 1 Control video.

10.7717/peerj.9610/supp-2 Supplemental Information 2 Magic video.

10.7717/peerj.9610/supp-3 Supplemental Information 3 All data.

## Additional Information and Declarations

### Data Availability

The following information was supplied regarding data availability: Raw data are available in the Supplemental Files.
